# 3D reconstruction from 2D multi-view dental 2D images based on EfficientNetB0 model

**DOI:** 10.1038/s41598-025-12861-3

**Published:** 2025-08-06

**Authors:** Waleed Mohamed, Nermeen Nader, Yasmin M. Alsakar, Naira Elazab, Mohamed Ezzat, Mohammed Elmogy

**Affiliations:** 1 Computer Science Department, Faculty of Computers and Information, Mansoura University, Mansoura, Dakahlia 35516 Egypt; 2 Information Technology Department, Faculty of Computers and Information, Mansoura University, Mansoura, Dakahlia 35516 Egypt; 3 Directorate of Health in Dakahilia, Ministry of Health and Population, Mansoura, Egypt

**Keywords:** Dentistry, 3D reconstruction, 3D LSTM, EfficientNetB0, 2D multi-view dental images, Electrical and electronic engineering, Biomedical engineering, Mathematics and computing

## Abstract

Dental diseases are the primary cause of oral health concerns around the world, affecting millions of people. Therefore, recent developments in imaging technologies have transformed the detection and treatment of oral problems. Applying three-dimensional (3D) reconstruction from two-dimensional (2D) dental images, such as X-rays, is a potential development field. 3D reconstruction technology converts real-world goals into mathematical models that are compatible with computer logic expressions. It’s been commonly used in dentistry. Particularly for patients with a vomiting reflex, 3D imaging techniques minimize patient discomfort and shorten the length of the examination or treatment. Therefore, this research paper proposes a new 3D reconstruction model from 2D multi-view dental images. The proposed framework consists of three stages. The first stage is the encoder stage, which extracts meaningful features from the 2D images. The second stage captures spatial and semantic information essential for the reconstruction task. The third stage is recurrence, which uses 3D long short-term memory (LSTM). It ensures that the information from various viewpoints is effectively integrated to produce a coherent representation of the 3D structure and decoder stage to translate the aggregated features from the LSTM into a fully reconstructed 3D model. When the proposed model was tested on the ShapeNet dataset, the suggested model achieved a maximum intersection over union (IoU) of 89.98% and an F1_score of 94.11%. A special case of 3D reconstruction, a dental dataset, has been created with the same structure as the ShapeNet dataset to evaluate our system. The proposed approach’s results show promising accomplishments compared to many state-of-the-art approaches, and they motivate the authors to make plans for further improvement.

## Introduction

Dentistry has rapidly changed over the previous several decades due to technological advancements in treatment and diagnosis^1–4^. Today, medical imaging is considered crucial for detecting and treating disorders in all medical professions^5^. Numerous medical imaging techniques are available for diagnosis, such as optical coherence tomography (OCT), laser-based pens for cavity detection, and X-ray radiography^6,7^. Dental diagnostic X-ray imaging is still one of the most widely used radiological modalities for oral examinations in dental clinics. It is essential for identifying and treating dental disorders, comprehending their nature, and anticipating them in their early stages. However, even the most experienced dentists may find that analyzing dental X-rays is a laborious and error-prone process that often leaves out important details. Therefore, using dental X-ray images to recreate 3D models will be beneficial^8–12^.

3D reconstruction has become one of the most popular areas of computer vision research, which accurately restores objects’ three-dimensional properties using their two-dimensional features. The human visual system is simulated via 3D reconstruction, which gives computers eyes representing transmitters and brains representing algorithms^13–15^. It also converts actual objectives into mathematical models compatible with computer logic expressions, and it has been used extensively in dentistry^5,16,17^. Researchers have worked intensively on advancing methodologies for 3D reconstruction from 2D X-ray images over the past decades^18–23^. In addition to reducing radiation exposure and patient expenses, these efforts seek to provide better details and greater precision^24^. Due to deep learning’s notable successes in fields connected to computer vision, scientists are now looking at how it may be used for 3D reconstruction from 2D X-rays.

The capability of 3D reconstruction to capture, replicate, process, analyze, and comprehend both static and dynamic images of dental procedures in real time. It offers the potential to more accurately describe or diagnose each disease and conduct a more in-depth analysis^25^. 3D images are a powerful tool for communication. A patient or physician can manipulate it to interactively view the scan target from any perspective. Furthermore, it improves the accuracy and efficiency of communication in remote situations using 3D images. 3D reconstruction technology is commonly employed in orthodontics, restorative dentistry, cranial bone, surgical navigation, and other areas of dentistry^26,27^. Even though 3D reconstruction technology has challenges, such as operator skill, inconsistent scanner quality, and high initial expenses, it has been crucial to dental care. The dental field has significantly transformed due to the digital revolution^28^.

Therefore, this research paper proposes a new method for 3D object reconstruction from 2D images using EfficientNetB0 deep convolutional neural network. As a result, the following are the primary contributions of the suggested method:

- Developing a dental dataset for 3D reconstruction, namely TeethNet, with the same structure as the ShapeNet dataset.
- Enhancing the 3D reconstruction performance by using the benefits of EfficientNetB0 within the encoder stage.
- Different evaluation metrics have been applied to the developed TeethNet dataset, such as mean absolute error (MAE), mean square error (MSE), and root mean squared error (RMSE).

The remainder of this research is structured as follows.  “Related work” shows the previous studies that discussed the 3D reconstruction, including their techniques. “The proposed framework” presents the proposed framework, including the description of each stage used to reconstruct the 3D model from the 2D dental image. The experimental results are shown in “Experimental results”, which explains the datasets and evaluation metrics used and then moves on to the obtained results and their analysis. Lastly, “Conclusion” presents the conclusion and future work.

## Related work

The topic of 3D reconstruction from 2D images has been the subject of numerous studies, utilizing improvements in machine learning and computer vision techniques^29–33^. Traditional methods often relied on geometric approaches, such as structure-from-motion (SfM) and stereo vision, which utilized numerous views of the same scene to estimate depth and create 3D models. These methods demonstrated success in controlled environments but faced challenges in dealing with noise, textureless regions, and varying lighting conditions. More recently, deep learning techniques have revolutionized 3D reconstruction by enabling single-view and multi-view reconstructions using convolutional neural networks (CNNs) and transformer-based models. These methods have been employed in different fields, including cultural heritage preservation and robotics, demonstrating their ability to capture fine-grained features and complex geometries. Furthermore, hybrid methods that combine geometric and deep learning models have emerged to enhance reconstruction accuracy and robustness. Despite significant progress, challenges remain in achieving real-time performance, handling large-scale scenes, and generalizing to unseen environments. This section discusses some research for applying 3D construction from 2D images.

Liang et al.^34^ presented a method for applying 3D teeth reconstruction from X-rays, which was particularly important for diagnosing dental disorders and performing several clinical procedures. This method created 3D teeth from a single image using the ConvNet network, which divides the task into teeth localization and single-shape estimation. This method achieved the highest performance in computing the 3D structure of the cavity and reflected the tooth details with 68.1%. Chen et al.^35^ presented a method that reconstructs 3D teeth from five intra-oral images to help visualize the patients’ conditions in virtual consultations. This method used images to recreate upper and lower tooth shape, arrangement, and occlusion. The modified U-net network used an iterative process that alternated between finding point correspondences and optimizing a compound loss function to match the parametric teeth model to expected tooth outlines. Their dataset was split using five-fold cross-validation. This method achieved 76.72% for the Dice similarity coefficient.

Ali et al.^36^ proposed a method for producing a 3D teeth crown using a prototype intraoral custom design and public software. Several images of the unusual teeth were taken from various perspectives. Scale-invariant feature transform (SIFT) was used to extract key points from images. The RANSAC matching algorithm was applied, and then motion formed the structure. The camera posed and a 3D point cloud was computed. Finally, a 3D object was visualized and was created. The error was computed ranged from 0.003%: 0.13%. Farook et al.^37^ provided a method for producing 3D dental teeth for digital partial dental crown synthesis and validation. To construct partial dental crowns (PDC), this method utilized a 3D-CNN. This approach was 60% accurate.

Minhas et al.^38^ developed a 3D teeth reconstruction approach to assess the position of maxillary impacted canines using panoramic X-rays. The information was compiled from CBCT scans of 123 patients, including 74 with impacted canines and 49 without. Using 3D Convolution Neural Network (3D CNN), this method produced a mean structure similarity index measure (SSIM) of 0.71 and an accuracy of 55%. Song et al. ^39^ suggested an approach for reconstructing 3D teeth from 2D images. This solution relied on Oral-3Dv2 to handle the cross-dimensional translation challenge in dental healthcare by learning exclusively from projection data. By converting 2D coordinates into 3D voxel density values, this model was trained to depict 3D oral structures. It produced 86.04% performance in 3D reconstruction from a single panoramic X-ray image.

Li et al.^40^ developed a 3D oral image reconstruction method. It used a Multilayer Perceptron (MLP) CNN pyramid network (3DPX) to reconstruct oral PX from 2D to 3D. The 3DPX method combines CNNs and MLPs to capture long-range visual dependency. It achieved 15.84 % PSNR, 63.72% DSC, and 74.09% SSIM. Ma et al.^41^ offered a technique for converting a single panoramic X-ray into 3D point cloud teeth. Using a two-stage framework and a single PX image, PX2Tooth was utilized to reconstruct 3D teeth. First, permanent teeth were segmented from PX images using the PXSegNet, which provided morphological, positional, and categorical information for every tooth. Then, to convert random point clouds into 3D teeth, a unique tooth-generating network (TGNet) was created. In terms of intersection over union (IoU), this approach obtained 79.3%. Choy et al.^42^ suggested a 3D recurrent reconstruction neural network (3D-R2N2) model. One or more images of a dataset taken from different angles were used to teach this network. It produces the object reconstruction from these images and does not require object class labels or image annotations for training or testing. Their experimental research revealed that their reconstruction framework outperformed cutting-edge single-view reconstruction approaches.

This section reviews several studies on applying 3D reconstruction from 2D images for various objects, including teeth, chairs, pens, cars, and more. However, these studies face several limitations. A key challenge is their reliance on specific datasets, which restricts the generalizability of the methods to diverse real scenarios. Additionally, many approaches encounter issues such as operating with a limited number of images, handling low-contrast images, and achieving low accuracy. Moreover, one major obstacle to advancement in this discipline is the lack of publicly accessible datasets for 3D reconstruction from 2D images.

Using the ShapeNet public dataset, a technique for 3D reconstruction from 2D images taken from various perspectives has been implemented to overcome the aforementioned difficulties. This proposed methodology enhances the reconstruction process by leveraging a multi-stage framework. The first stage involves an encoder extracting meaningful features from the 2D images, capturing spatial and semantic information essential for the reconstruction task. In the second stage, a recurrent network utilizing 3D long short-term memory (LSTM) is employed to model the temporal and spatial dependencies between the multiple views. This stage ensures that the information from different perspectives is effectively integrated to produce a coherent representation of the 3D structure. Finally, the third stage consists of a decoder that translates the aggregated features from the LSTM into a fully reconstructed 3D model. This comprehensive pipeline not only improves the quality of the reconstructed 3D object but also addresses issues related to variability in object shape, viewing angles, and image consistency. Using a publicly available dataset like ShapeNet further enhances the reproducibility and comparability of the results, making this approach a significant step forward in 3D reconstruction. Table 1 discusses previous papers according to year, methodology, metrics, strengths, and limitations.

**Table 1:** The comparison of the related studies of the 3D construction based on 2D images.

Paper	Methodology	Metric	Strengths	Limitations
Liang et al.^34^	ConvNet X2Teeth	IOU: 0.681	Precise reconstruction with tooth details	Worked on single image
Chen et al.^35^	parametric, statistical, and U-net model	Dice: 0.7672	Good in dental monitoring	worked on only five images
Ali et al.^36^	SIFT and RANSIC	Error: 0.003 : 0.131	Important teeth crown details	Worked on SIFT descriptor only
Farook^37^	3D-CNN	Accuracy: 0.60	Provided the teeth textural details	Low accuracy
Minhas^38^	3D-CNN	Accuracy:0.55	Worked on any images number	Low accuracy
Song et al.^39^	Multi-head neural X-ray	Accuracy: 86.04%	Good complexity analysis	Small dataset
Li et al.^40^	(MLP)-CNN and 3DPX	PSNR: 15.84 DSC: 63.72 SSIM: 74.09	Enhanced reconstruction quality	Low contrast images
Ma et al.^41^	PX2Tooth network	IOU: f 0.793	Provided the teeth textural details	Low IOU
Choy et al.^42^	3D-R2N2	IOU: 0.634	Dealing with images with insufficient texture	Low IOU

## The proposed framework

**Fig. 1:**
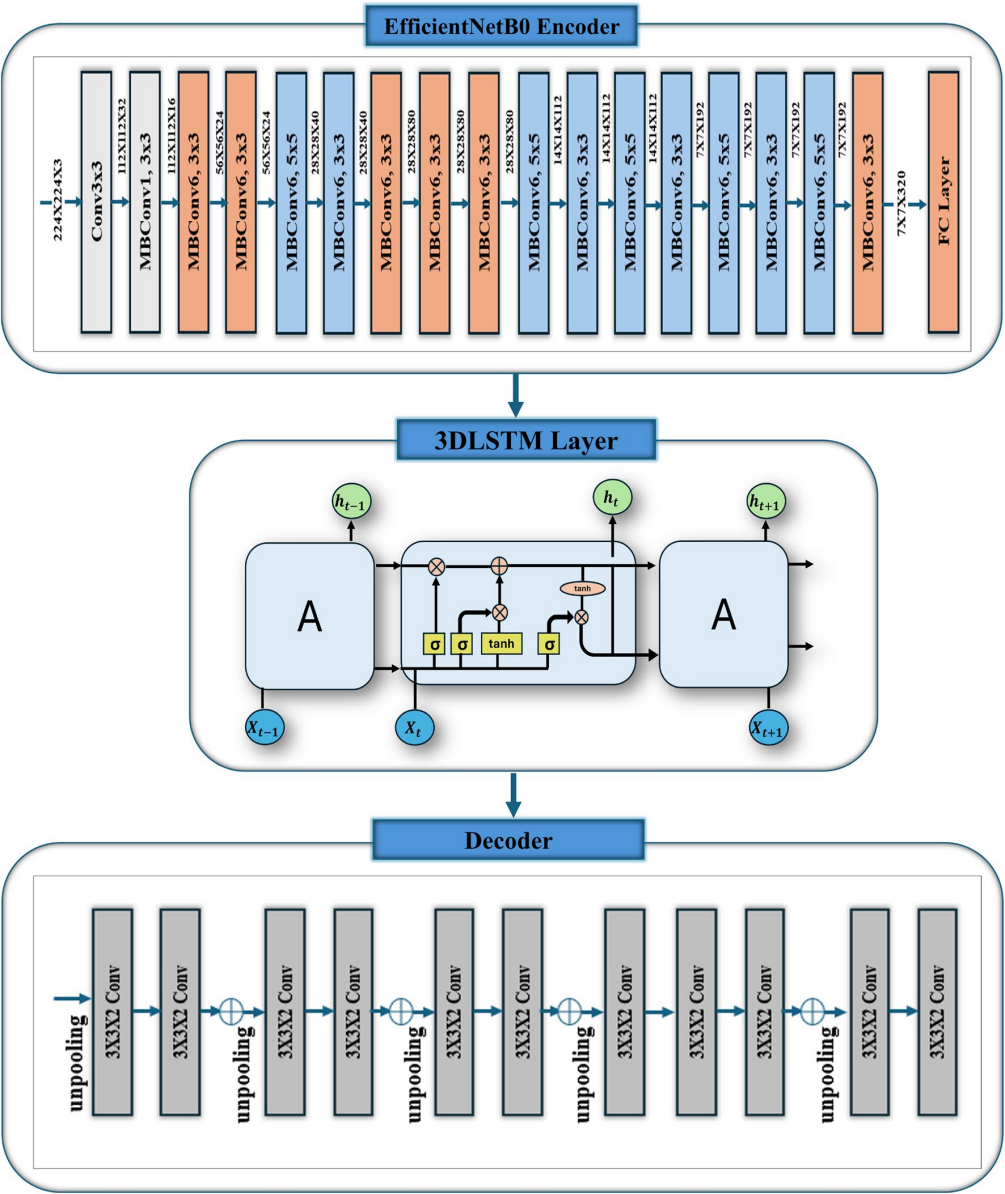
The 3D reconstruction from 2D images based on EfficientNetB0 encoder, LSTM, and decoder.

The proposed work aims to design and train an advanced 3D object recognition model using a combination of multiview RGB images and voxel representations from the ShapeNet dataset as indicated in Fig. 1. The model’s architecture integrates state-of-the-art techniques in deep learning, combining EfficientNetB0 for 2D feature extraction, Conv3D LSTM cells for temporal learning of 3D structures, and 3D deconvolution for voxel reconstruction. Therefore, in this work, no explicit calibration has been performed in the traditional sense. Instead, we utilize a deep neural network trained on multiview images, where the network inherently learns spatial consistency and scale relationships from the training data^42,43^.

### Encoder

The proposed encoder leverages the EfficientNet-B0 architecture to extract robust 2D feature representations^44,45^. EfficientNet-B0, pre-trained on a large dataset, serves as the backbone, and its convolutional layers up to the penultimate stage are utilized for feature extraction. The classification head of the pre-trained model is excluded, ensuring that only spatially rich features are retained. These feature maps are flattened into a 1D vector during the forward pass to prepare for further processing.

**Fig. 2:**
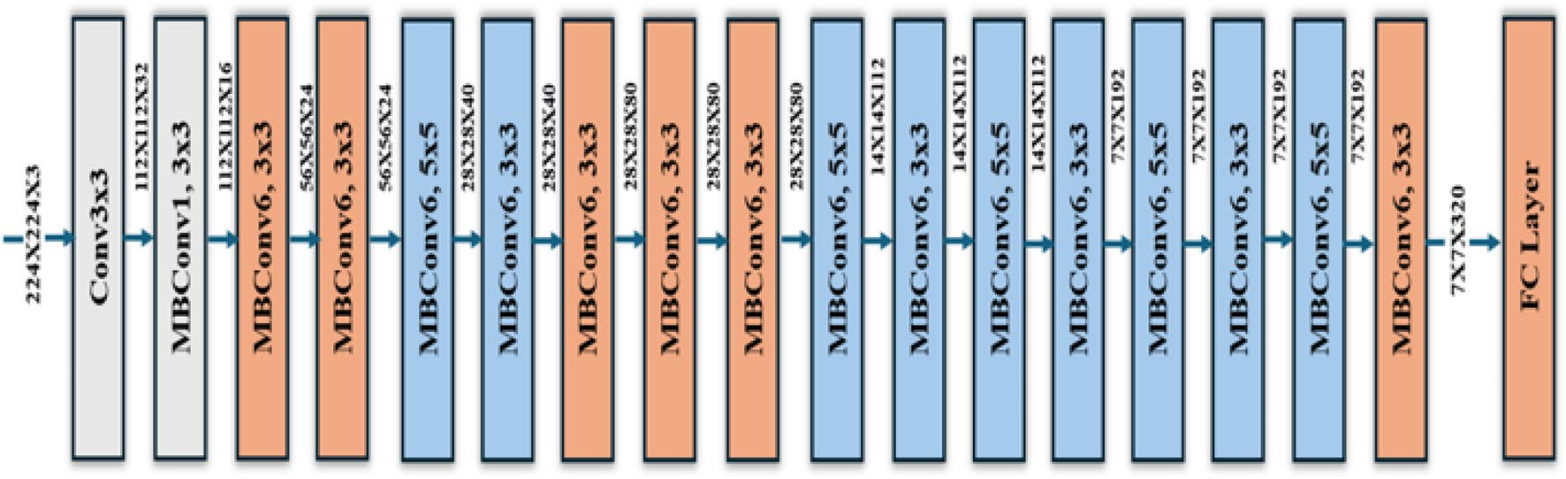
The encoder architecture based on the EfficientNetB0 model.

To adapt to varying input dimensions, the encoder dynamically initializes a fully connected layer based on the flattened feature map’s size, determined during the first forward pass. This fully linked layer reduces dimensionality to a fixed 1024-dimensional latent space, assuring compatibility with subsequent tasks. A LeakyReLU activation function with a negative slope of 0.1 is used to introduce nonlinearity and reduce concerns with vanishing gradients. The encoder design combines EfficientNet-B0’s efficient feature extraction with a flexible and computationally efficient architecture, making it well-suited for diverse applications requiring compact yet powerful feature representations. Fig.2 presents the encoder architecture.

### Recurrence: 3D LSTM

**Fig. 3:**
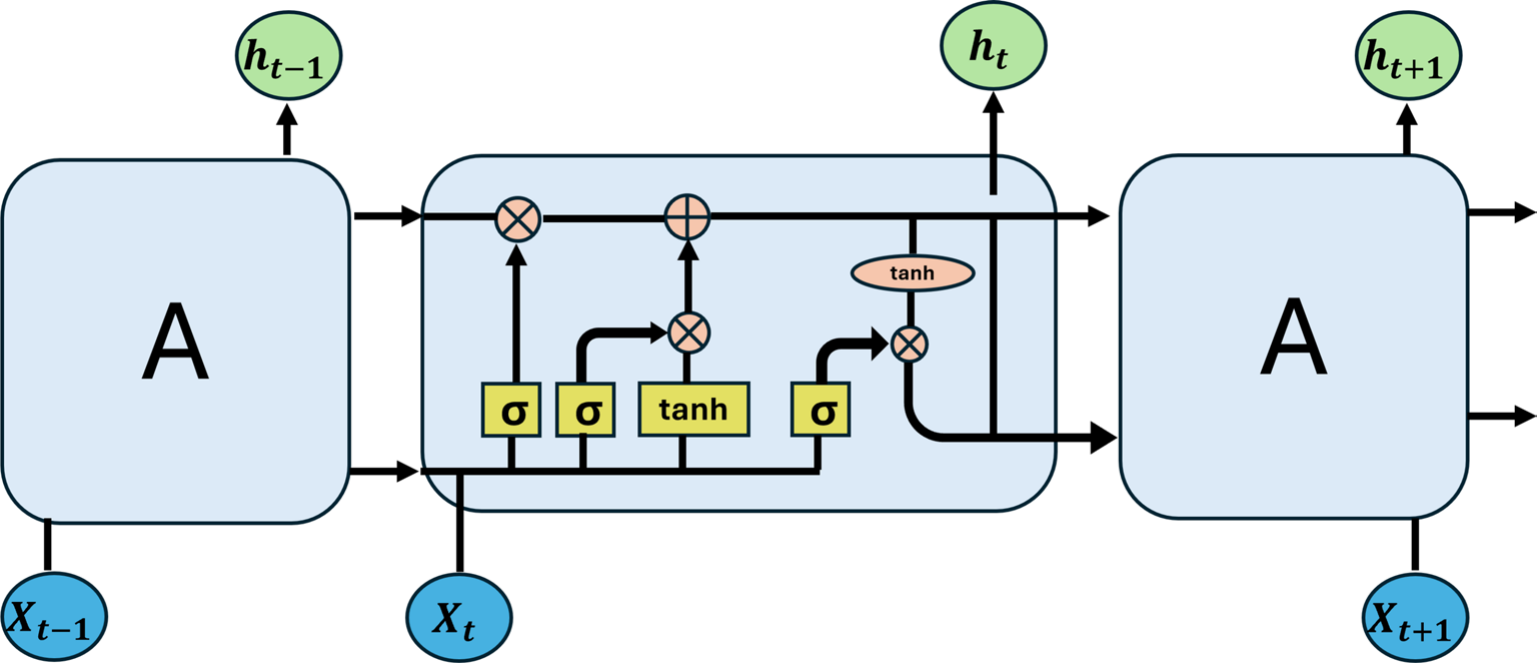
The 3D LSTM architecture.

Our 3D-R2N2’s core component is a recurrence module, which allows the network to remember what it has already seen and update its memory whenever a new image is discovered. A three-dimensional convolutional LSTM (3D-LSTM) has been proposed. The network is composed of well-organized LSTM units with minimal connectivity. As depicted in Fig. 3, the 3D-LSTM units are dispersed in a grid pattern, each reproducing a fraction of the outcome. The 3D grid has $N$ $\times$ $N$ $\times$ N 3D-LSTM units, where N is the grid’s spatial resolution. Each 3D-LSTM unit (indexed $(i, j, k)$, has an independent hidden state $h_t(i, j, k)$ $\in$ $R^{N_h}$. The equations that regulate the 3D-LSTM grid are the following:

1 $$\begin{aligned} & f_t = \sigma \left( W_f T(x_t) + U_f *h_{t-1} + b_f \right) \end{aligned}$$

2 $$\begin{aligned} & i_t = \sigma \left( W_i T(x_t) + U_i *h_{t-1} + b_i \right) \end{aligned}$$

3 $$\begin{aligned} & s_t = f_t \odot s_{t-1} + i_t \odot \tanh \left( W_s T(x_t) + U_s *h_{t-1} + b_s \right) \end{aligned}$$

4 $$\begin{aligned} & h_t = \tanh (s_t) \end{aligned}$$

where $s_t$ is the state at time t, $f_t$ is the forget gate, and $s_{t-1}$ is the previous state, and $i_t$ is the input gate.

### Decoder

**Fig. 4:**
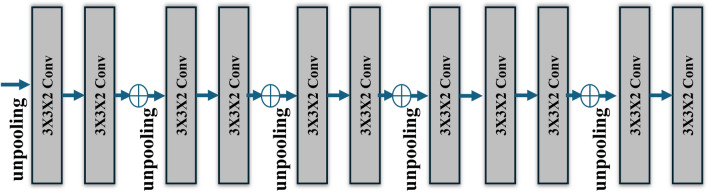
The decoder architecture.

After obtaining the input image sequence $x_1$, $x_2$,..... $x_T$, the 3D-LSTM employs 3D convolutions, nonlinearities, and unspooling to enhance the resolution of the hidden state $h_T$. As illustrated in Fig. 4, a basic decoder network with five convolutions and a deep residual version with four residual connections, followed by a final convolution, has been presented that is comparable to our encoders. After reaching the intended output resolution, a voxel-wise softmax was used to transform the final activation to the occupancy probability of the voxel cell.

## Experimental results

The performance of the proposed scheme for 3D construction from 2D images was evaluated using Python, a highly versatile and user-friendly programming language renowned for its extensive library support. Python is used extensively in domains like artificial intelligence and bioinformatics. For this study, we leveraged several libraries, including NumPy, Matplotlib, TensorFlow, Scikit-learn, Keras, and OpenCV, to develop and evaluate the models effectively. The computer configuration used in these experiments is as follows: CPU: Intel(R) Core(TM) i7-9750H @ 2.60 GHz (Lenovo, Beijing, China); Memory: 16 GB RAM; Operating System: Microsoft Windows 10 (Microsoft, Redmond, WA, USA); Programming Environment: Python. In the next section, we detail the metrics used for performance evaluation.

### Dataset description

**Fig. 5:**
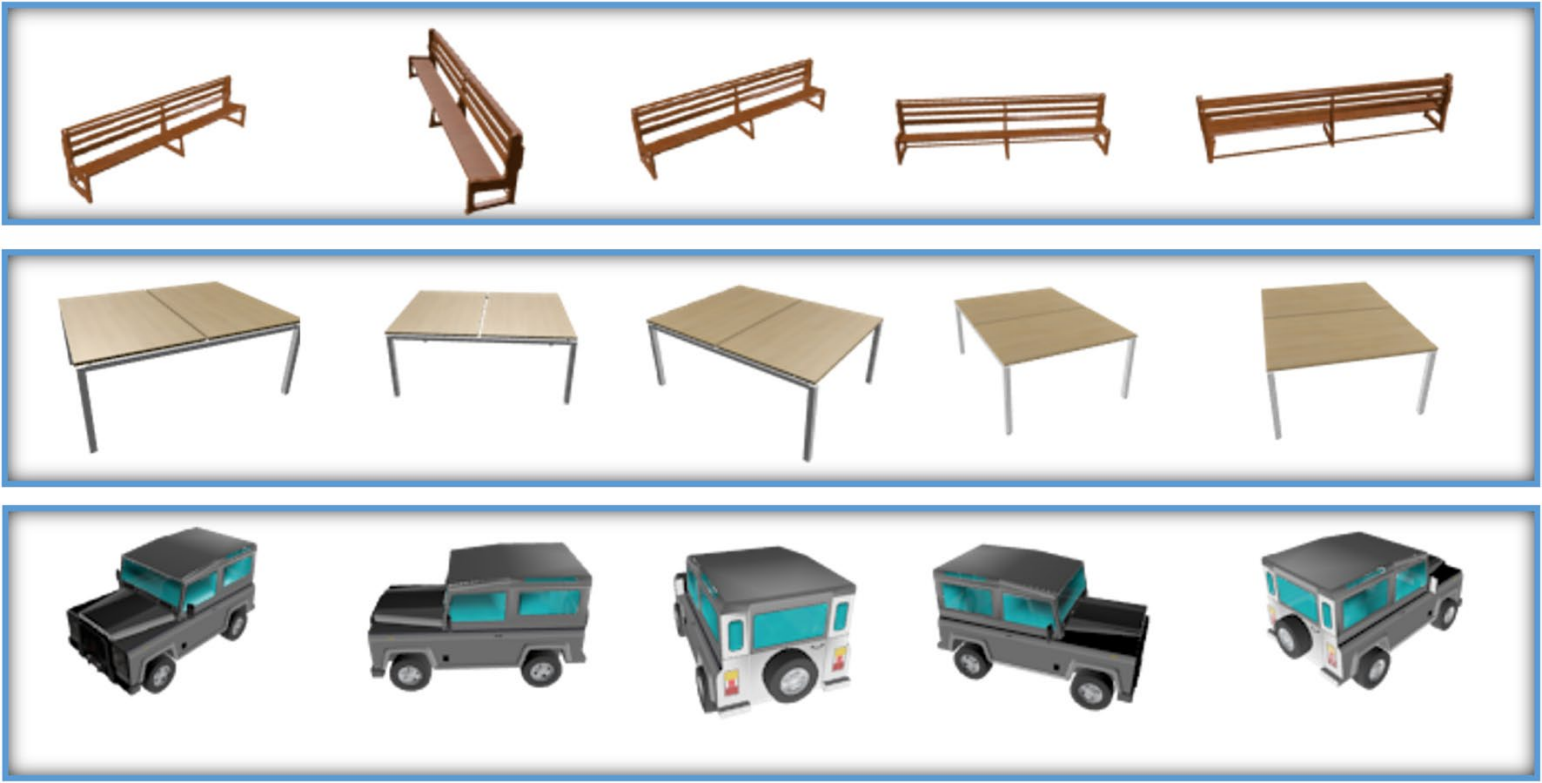
The ShapeNet dataset images samples.

The ShapeNet dataset, which comprises 3D CAD models organized following the WordNet hierarchy, was used to evaluate the suggested approach. Thirteen main categories and 50 thousand models were used from a portion of the ShapeNet dataset^42^. Fig. 5 displays a selection of the ShapeNet dataset images.

#### The case study dataset

**Fig. 6:**
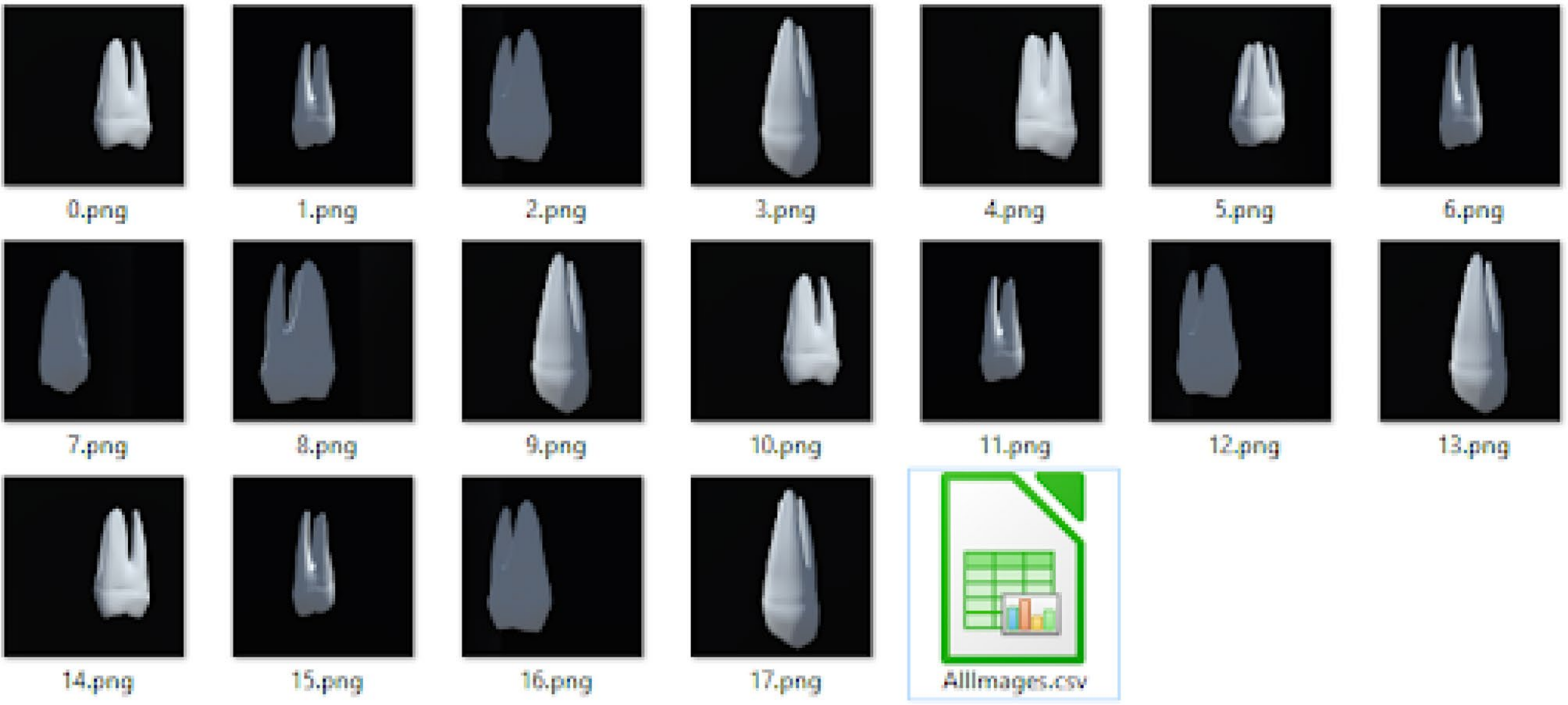
The generated image dataset sample contents.

Due to a lack of teeth X-ray datasets online, the team started to create a graphical dataset simulation for the teeth dataset, namely TeethNet, to be used for initial testing. The generated multiview dataset has been loaded in GitHub repository^46^. The dataset contains the following as shown in Fig. 6:

- Thirty-two folders (each folder is named after the corresponding tooth iso name) contain 18 images of different views generated by Unity 3D C# scripts.
- Ground truth OBJ 3D format: 3D format for each tooth as Mesh OBJ files. It contains 32 models.
- Ground truth Binvox 3D format: 3D voxel format for each tooth. It contains 32 models.

The last thumbnail in Fig. 6 shows a CSV (comma-separated values) file that contains the image names list and their relative positions during the generation of the dataset. The relative position is computed with respect to the first image in the dataset. Also, a few samples look like 2D while others look like 3D as all images are 2D projections based on the viewer’s location and angle. In some cases, the viewer’s angles have a visual appearance of 3D shades depending on light source reflection differences in the simulated environment. However, in other images, the angle of view has almost equal light reflections, giving a 2D-like visual appearance.

**Fig. 7:**
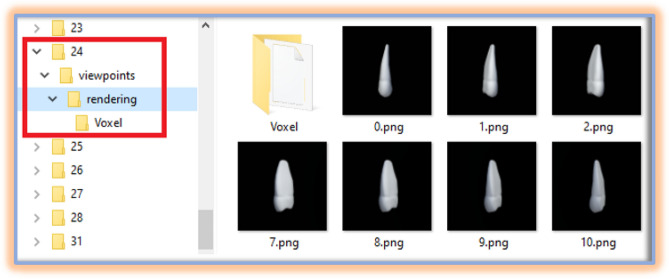
The directory structure of generated TeethNet dataset.

This dataset is developed using Unity 3D. Our custom dataset (TeethNet) has a metadata structure similar to the original ShapeNet dataset, as shown in Fig. 7.

### Evaluation metrics

Various evaluation measures can be employed to evaluate the performance of the 3D reconstruction system, such as IoU, F1 score, MAE, MSE, and RMSE. The voxel IoU between a 3D voxel reconstruction and its ground truth voxelized model can be computed by Eq.  5^42^.

5 $$\begin{aligned} IOU=\sum _{i,j,k}[I(p_{(i,j,k)}>t) I(y_{(i,j,k)}]/\sum _{i,j,k}[I(I(p_{(i,j,k)}>t)+ I(y_{(i,j,k)}))] \end{aligned}$$

where $(i,j,k)$ represents voxel coordinates, $t$ is voxelization threshold and the corresponding ground truth occupancy is $(y_{(i,j,k)}$ $\in$ ${0,1}$.

The F1 score is a prominent metric that measures the overall volume agreement between predicted and ground-truth 3D reconstructions^47^. MAE calculates the average magnitude of errors between expected and actual values, regardless of their direction^48^. It can be computed by Eq. 6.

6 $$\begin{aligned} \text {MAE} = \frac{1}{n} \sum _{i=1}^{n} |y_i - \hat{y}_i| \end{aligned}$$

where $y_i$ represents the actual value, $\hat{y}_i$ is the predicted value, and $n$ is the total number of samples.

MSE evaluates the average squared difference between the predicted and actual values^49,50^, penalizing larger errors more significantly than MAE. The formula for MSE can computed by Eq. 7. RMSE, or the square root of MSE, is an interpretable error measure in the same units as the original data^48,51^. It can be computed by Eq. 8.

7 $$\begin{aligned} \text {MSE} = \frac{1}{n} \sum _{i=1}^{n} (y_i - \hat{y}_i)^2 \end{aligned}$$

8 $$\begin{aligned} \text {RMSE} = \sqrt{\frac{1}{n} \sum _{i=1}^{n} (y_i - \hat{y}_i)^2} \end{aligned}$$

The Chamfer distance (CD) is used to quantify the difference between two point clouds by computing the squared distance between each point $p$ in the predicted point cloud $X_p$ and its closest neighbor in the true point cloud $X$, and vice versa. It is formulated as follows:

9 $$\begin{aligned} CD = \sum _{p \in X_p} \min _{q \in X} \Vert p - q \Vert ^2 + \sum _{q \in X} \min _{p \in X_p} \Vert p - q \Vert ^2 \end{aligned}$$

The lower (denoted by $\downarrow$) the CD is, the better the reconstructed 3D shape is. The Earth Mover’s Distance (EMD) quantifies the dissimilarity between two multi-dimensional distributions in a given feature space by computing the point-to-point $L_1$ distance between two point clouds. It is defined as:

10 $$\begin{aligned} \text {EMD} = \min _{\phi : X_p \rightarrow X} \sum _{p \in X_p} \Vert p - \phi (p) \Vert \end{aligned}$$

The lower (denoted by $\downarrow$) the EMD value, the better the quality of the reconstructed 3D shape.

### Results

This subsection presents and explains the results of the proposed system evaluated on the ShapeNet dataset.

#### A comparison of deep learning methods

Deep learning models such as EfficientNetB0, ResNet18, VGG16, and ResNet50 are well-known for their performance in various computer vision applications. EfficientNetB0 is notable for its revolutionary compound scaling method, allowing it to compromise accuracy and processing economy. ResNet18, with its lightweight architecture and residual learning framework, is particularly suited for tasks where computational resources are limited^52^. VGG16, characterized by its deep architecture with 16 layers, excels at capturing hierarchical features but requires higher computational power^53^. ResNet50, a deeper variant of the ResNet family, incorporates bottleneck blocks to efficiently learn complex representations, making it ideal for intricate tasks requiring detailed feature extraction^54^. These models collectively provide robust solutions for feature extraction and classification, each with unique strengths tailored to different application scenarios.

A comparison between the suggested system employing EfficientNet_B0 and other deep learning techniques, such as Resnet18, VGG19, and Resnet50, has been provided, as indicated in Table 2. This comparison demonstrates the superior outcome of the suggested work, which can be attributed to the parameter efficiency of EfficientNet_B0, which enables the model to achieve high accuracy without incurring excessive computational costs, and its compound scaling strategy, which optimally balances depth, width, and resolution. Furthermore, EfficientNet’s ability to extract rich features and effectively handle spatial information, combined with its training efficiency and generalization capabilities, make it ideal for complex tasks such as 3D reconstruction, where accuracy and computational efficiency are critical.

**Table 2:** A comparison of the proposed system using EfficientNet_B0 and other deep learning methods on ShapeNet dataset.

Network	IOU	F1_Score
Resnet18	62.10%	74.52%
VGG16	65.78%	76.26%
Resnet50	64.19%	75.13%
MobileNet	63.88%	74.95%
InceptionResnet	67.13%	77.40%
**Proposed work**	**89.98**%	**94.11**%

Significant values are in bold.

#### A comparison of different versions of EfficientNet

The comparison of different EfficientNet variants as encoders, as shown in Table 3, demonstrates the impact of model complexity on the reconstruction performance. EfficientNetB0 achieved the highest IoU of 89.98% and an F1-score of 94.11%, outperforming EfficientNetB1 and EfficientNetB2. This superior performance suggests that EfficientNetB0 balances capturing spatial-semantic features effectively and maintaining computational efficiency. Also, EfficientNet-B1 and B2 have a larger number of parameters, which may have led to overfitting, particularly given the dataset size. Reconstructing 3D structures from 2D images requires efficient feature extraction while maintaining fine-grained details. Our results suggest that B0’s lighter architecture is sufficient for capturing the necessary spatial features, while deeper architectures might introduce unnecessary complexity without significant benefits. These results highlight the effectiveness of EfficientNet_B0 as the encoder stage in the proposed framework.

**Table 3:** A comparison of different versions of EfficientNet on ShapeNet dataset.

Network	IOU	F1_Score
EfficientNetB0	89.98%	94.11%
EfficientNetB1	82.84%	88.68%
EfficientNetB2	83.04%	89.24%

#### A comparison with the state-of-the-art approaches

Table 4 presents a comparative analysis of recent state-of-the-art approaches for 3D reconstruction on the ShapeNet dataset. It includes studies from 2016 to 2025, listing the research authors, network architectures, and performance metrics such as Intersection over Union (IOU) and F1-score. The table effectively highlights the progression of methods, from early architectures like 3D-R2N2 Choy et al.^42^ to more advanced models such as Multi-Manifold Attention kalitsios et al.^55^. Notably, some entries have missing values, such as the IOU score for liu et al.^56^ and the F1-score for Choy et al. (2016), which may indicate either unreported metrics or inapplicability to those models. Additionally, the proposed methodology (EfficientNetB0, 2025) achieves the highest IOU (89.98) and F1-score (94.11), suggesting significant improvements over previous methods. However, the formatting of the table could be refined for better readability, particularly in aligning numerical values and handling long network names that span multiple lines. The highlighted title effectively draws attention but may need reformatting for consistency with academic standards. Overall, the table provides a clear comparison of different approaches, emphasizing the advancements in 3D reconstruction techniques over time.

**Table 4:** The proposed framework results compared to the recent state-of-the-art approaches on the ShapeNet dataset.

Year	Research	Network	IOU (%)	F1-score (%)	CD (%)	EMD (%)
2016	Choy et al.^42^	3D-R2N2	63.4	–	–	–
2021	Yang et al.^57^	LSTM shape encoder	38.7	14.3	–	–
2024	Kalitsios et al.^55^	Multi-manifold attention	78.12	56	–	–
2025	Liu et al.^56^	Prior-guided adaptive probabilistic network for single-view 3D reconstruction	–	54.42	2.62	5.99
2025	**Proposed methodology**	**EfficientNetB0**	**89.98**	**94.11**	**0.41**	**0.24**

Significant values are in bold.

#### Computing 3D Euler angle based on features descriptors

The provided code implements a pipeline for extracting Euler angles and reconstructing 3D structures from 2D image pairs^58–60^. Keypoint detection and matching are central to this process^61–64^, as they help establish correspondences between image pairs. The BRISK algorithm is utilized to detect and describe keypoints^65,66^, with the BFMatcher ensuring robust matching of descriptors. The fundamental matrix is computed using these correspondences, and the essential matrix is derived using the intrinsic camera matrix. The camera’s relative pose, including the rotation matrix R and translation vector t, is recovered from the essential matrix. Finally, the 3D points are triangulated, and their spatial arrangement is visualized in a point cloud^67,68^. This approach highlights the synergy of computer vision techniques and mathematical rigor to recover geometric transformations and compute Euler angles, which are essential for applications like robotics, augmented reality, and medical imaging.

**Fig. 8:**
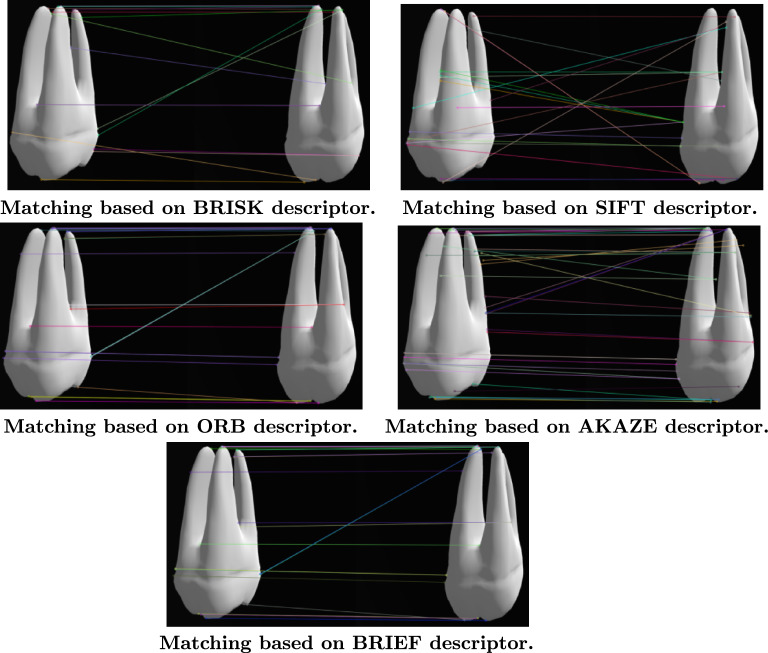
The matching results between dental images based on various descriptors.

Feature descriptors like binary robust independent elementary features like binary robust invariant scalable keypoints (BRISK), SIFT, oriented FAST and rotated brief (ORB), BRIEF, and AKAZE are essential components of computer vision and image processing. Each algorithm has distinct advantages depending on the application. For instance, SIFT excels in scale and rotation invariance, making it ideal for scenes with varying perspectives^69,70^. ORB offers a fast and efficient alternative with lower computational overhead, suitable for real-time tasks^71,72^. BRIEF emphasizes simplicity and speed by encoding binary descriptors^73,74^. Similarly, AKAZE focuses on nonlinear scale spaces, ensuring robustness against varying scales and affine transformations^75,76^. As used in the code, BRISK efficiently detects and describes features while ensuring robustness to rotation and scale changes^65,66^. These descriptors form the foundation of matching and reconstruction pipelines, enabling accurate spatial and geometric transformation recovery. Fig 8 indicates The matching results between dental images based on various descriptors.

To validate the accuracy of 3D reconstruction and pose estimation, the code computes key error metrics such as MAE, MSE, and RMSE. These metrics compare vectors, such as those derived from Euler angles or reconstructed 3D points, against ground truth values. MAE captures the average magnitude of errors, offering a straightforward measure of accuracy. MSE emphasizes larger errors due to its squared nature, providing insights into outlier behavior. RMSE, as the square root of MSE, presents the error in the same units as the original data, making it more interpretable. These metrics are crucial for assessing the precision and reliability of 3D reconstruction pipelines, particularly in applications like medical imaging, where even minor inaccuracies can lead to significant implications.

Table 5 presents the experimental results for various methods based on deep learning, focusing on keypoint detectors and descriptors, evaluated using three error metrics: MAE, MSE, and RMSE. Among the methods, the BRISK detector and descriptor achieved the lowest error rates, with an MAE of 0.087, MSE of 0.0083, and RMSE of 0.091, indicating superior accuracy and robustness. In contrast, SIFT, ORB, and BRIEF detectors and descriptors exhibit higher error rates, with identical MAE values of 0.37 and similar RMSE values of approximately 0.39, suggesting their performance is comparable but less precise than BRISK. AKAZE detector and descriptor performed better than SIFT, ORB, and BRIEF, with moderate error rates (MAE of 0.17, MSE of 0.044, and RMSE of 0.21), showcasing a balance between computational efficiency and accuracy. These results underline BRISK’s effectiveness in minimizing errors, making it a promising choice for deep learning-based applications requiring precise keypoint detection and description.

**Table 5:** A comparison for computing different error metrics for 3D Euler angle based on different features descriptors.

Method	MAE	MSE	RMSE
BRISK detector and descriptor	0.087	0.0083	0.091
SIFT detector and descriptor	0.37	0.19	0.43
ORB detector and descriptor	0.37	0.15	0.39
AKAZE detector and descriptor	0.17	0.044	0.21
BRIEF detector and descriptor	0.37	0.15	0.39

## Conclusion

3D reconstruction is crucial in advancing dental imaging by providing detailed and accurate representations of oral structures, enabling precise diagnostics and treatment planning. This technology enhances traditional 2D imaging methods by creating comprehensive models that improve visualization, reduce patient discomfort, and streamline clinical workflows. In conclusion, this research highlights the potential of advanced 3D reconstruction techniques to address challenges in dental imaging and diagnosis. By leveraging a robust framework comprising an encoder for feature extraction, a 3D LSTM for integrating multi-view information, and a decoder for generating the final 3D model, the proposed system demonstrates significant improvements in accuracy and coherence. The model’s performance, validated on both the ShapeNet dataset and a newly created dental dataset, underscores its effectiveness, achieving a maximum IOU of 89.98% and F1-Score of 94.11%. These results show the system’s potential for practical applications in dentistry, offering enhanced precision, reduced patient discomfort, and improved diagnostics and treatment planning efficiency. Future research will enhance the framework’s suitability for a wider range of dental and medical imaging scenarios, investigate the integration of extra modalities, and optimize it for clinical datasets.

## Data Availability

Two publicly accessible datasets were used to test this research study: ShapeNet Dataset (https://shapenet.org/)^42^. The Case Study Dataset (https://github.com/waleedmm/TeethNet-Dataset/tree/main)^46^.
